# Breast-Conserving Oncoplastic Surgery Stratification: Morbidity Retrospective Analysis and its Association with Procedure Complexity Level

**DOI:** 10.1245/s10434-025-17838-0

**Published:** 2025-07-20

**Authors:** Inês de Cardoso Dias, Henrique Ferreira Mora, Bárbara Peleteiro, José Luís Fougo

**Affiliations:** 1 Faculty of Medicine, University of Porto, Alameda Prof. Hernâni Monteiro, Porto, Portugal; 2 Department of Surgery and Physiology, Faculty of Medicine, University of Porto, Alameda Prof. Hernâni Monteiro, Porto, Portugal; 3 Breast Centre, Centro Hospitalar Universitário São João, Porto, Portugal; 4 EPI Unit, Institute of Public Health, University of Porto, Porto, Portugal; 5 Laboratory for Integrative and Translational Research in Population Health, University of Porto, Porto, Portugal

**Keywords:** Breast cancer, Breast conservative surgery, Oncoplastic breast surgery, Complications of breast cancer surgery, Surgical morbidity

## Abstract

**Introduction:**

Breast-conserving oncoplastic surgery (BCOS), in association with radiotherapy, is the state of the art in the surgical treatment of breast cancer. In this study, we aimed to systematize and validate a novel, four-level complexity classification system for BCOS and associate it with surgical morbidity.

**Methods:**

We conducted a retrospective, observational study of consecutive female patients who underwent breast-conserving surgery between August 2022 and January 2024 at our breast center. Descriptive statistics were used to summarize the main sample characteristics. The primary outcome was surgical morbidity associated with the novel four-level complexity classification category of surgery performed, computed through a logistic regression model.

**Results:**

Overall, 304 patients underwent the procedures of interest in this study. Surgery complexity levels 1, 2, 3, and 4 were performed in 28, 121, 114, and 41 patients, respectively. A total of 95 patients had complications, including infection, seroma, hematoma, dehiscence, or other complications. A total of 28 patients required re-interventions after definitive diagnosis. The odds of complications increased according to the surgery complexity level, independently of risk factors for complications and factors linked to the surgery type selection, even when considering only clinically relevant complications.

**Conclusions:**

We concluded that there is an association between morbidity and the complexity level of the surgery performed, with the most complex techniques being associated with higher rates of overall complications and the need for re-intervention, validating the need for a new stratification system for surgeries to improve patients’ quality of life.

Breast cancer is a leading health concern due to its high incidence. It is the most commonly diagnosed malignant tumor worldwide, with the majority of the disease burden occurring in females. Despite the increasing incidence, mortality has been decreasing.^1,2^

Globalization and an expanding economy may increase breast cancer incidence in developing (64–95%) and developed (32–56%) countries by 2040.^3^

The state of the art of breast cancer treatment encompasses several modalities, such as radiation therapy, primary and adjuvant systemic therapy, and a wide range of surgical techniques.^4^ Mastectomy continues to be the appropriate surgical solution for some patients.^5^ Nevertheless, breast-conserving surgery associated with radiation therapy has achieved equivalent, if not better, results,^6^ improving quality-of-life standards such body image and future perspectives over mastectomy.^7^

The increasing number of breast cancer survivors requires ongoing improvements in therapeutic strategies. Oncoplastic techniques have become increasingly recognized over time, with greater acceptance of their effectiveness and safety profile.^8^ These techniques integrate plastic surgery techniques with oncological breast-conserving surgery, aiming to provide safe oncologic treatment with favorable cosmetic results.^9–11^

Studies demonstrated improved overall survival in patients with early-stage breast cancer who underwent breast-conserving surgery followed by adjuvant radiotherapy, compared with patients who underwent mastectomy.^12–14^

Although severe morbidity after breast-conserving oncoplastic surgery (BCOS) is low, short- and long-term morbidity can be observed, including complications, the need for re-intervention, and disease recurrence. These events can alter the normal postoperative course and negatively impact patients’ quality of life. The incidence rate of long-term morbidity in BCOS was 25.5 per 100 patients per year, which was significantly higher when compared with conventional breast-conserving surgery, with a rate of 11.3 per 100 patients per year.^15^

There is a lack of standardized scoring systems for assessing morbidity after BCOS. Currently, there are several classifications of breast surgical techniques, with the most frequently cited being those proposed by Urban, Clough et al., Weber et al., and the American Society of Breast Surgeons (ASBrS).^16–19^ We found that existing classification systems are sometimes ambiguous in how they categorize specific procedures and therefore do not ensure the reproducibility of their application.

In this study, we proposed a novel BCOS classification system, based on surgical complexity, that includes both non-oncoplastic and oncoplastic techniques, for the treatment of primary and locally recurrent breast cancer. The newly proposed classification seeks to systematize the multiplicity of conservative surgical techniques, organize them methodologically and stratify them based on the complexity of related procedures. This approach facilitates the precise and reproducible delineation of which interventions correspond to each defined complexity level. We aimed to study the occurrence of complications after surgery according to the level of surgical complexity, considering this classification system, to validate its applicability.

## Methods

We conducted an observational, retrospective study based on the registry of 304 consecutive patients. This sample size was determined based on the assumption that oncoplastic surgeries would present twice the risk of developing complications in the short term compared with conservative surgeries performed without the use of oncoplastic techniques, and predicting a complication rate in the latter group of 10–15%, for a significance level of 5% and a sample power of 80%. Inclusion criteria were female breast cancer patients aged ≥18 years who underwent BCOS, both in an outpatient and inpatient setting, at the Breast Centre of Unidade Local de Saúde de São João (ULSSJ) between August 2022 and January 2024.

This study was approved by the Ethics Committee of ULSSJ (project no. 203/2024). Patients’ informed consent was waived due to the retrospective design of this study.

Patient and tumor characteristics were collected through review of the patients’ clinical charts.

The variables collected included age at the time of surgery, major comorbidities such as body mass index (BMI), diabetes, hypertension, and smoking status. Additionally, data on patients’ history of previous breast surgeries and exposure to radiotherapy, chemotherapy, or hormonal therapy were collected. Anthropometrical data such as breast size, breast cup size, ptosis, BMI, and tumor characteristics at presentation (imaging and microbiopsy) were also documented, including palpable mass, size, location of the tumor, and radiological appearance. Furthermore, data regarding adjuvant and neoadjuvant treatments and tumor characteristics after surgery, as well as data regarding the surgery itself, the type of surgery according to complexity, duration of the surgery, need for hospitalization, and hospitalization time were also collected.

Patients were grouped according to the complexity level of the surgery they underwent. Our cohort was drawn from a high-volume, European Society of Breast Cancer Specialists (EUSOMA)-certified breast cancer center in Portugal. All procedures were performed by general surgeons with formal board certification in oncologic surgery and specialized training in oncoplastic breast surgery.

**Fig. 1:**
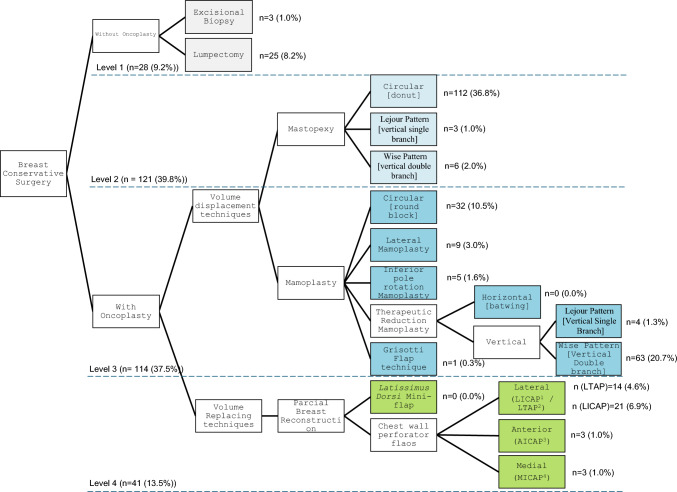
Surgery techniques stratification system, according to the level of complexity of execution. *LICAP* lateral intercostal artery perforator flap, *LTAP* lateral thoracic artery perforator flap, *AICAP* anterior intercostal artery perforator flap, *MICAP* medial intercostal artery perforator flap

Several breast-conserving surgery techniques were categorized in a novel four-level classification system, which comprises the surgical techniques mostly used in this breast center and that reflects the execution complexity level. The first level (level 1) encompasses techniques that do not require oncoplasty, such as excisional biopsy and lumpectomies. The second level (level 2) comprises mastopexy techniques, and the third level (level 3) comprises mammoplasty techniques. A mastopexy was considered when it involved minimal glandular tissue mobilization, typically in cases where <10% of breast volume was excised and where, at most, minimal local advancement glandular flaps were used to fill the defect. Procedures that required more extensive excisions, necessitating breast reduction or true parenchymal reshaping, were considered mammoplasties. Finally, volume replacement techniques for partial breast reconstruction were considered in the fourth level (level 4) (Fig. 1).

The occurrence of complications, type of complications (categorized according to the Clavien–Dindo classification), and reoperations due to tumor-involved margins or complications were registered. Furthermore, locoregional and distant disease recurrences, need for re-interventions, months of follow-up, and vital status were recorded. Our primary outcome was the occurrence of complications after surgery according to the level of surgical complexity, considering this classification system. All data collected were stored in a database in a completely pseudo-anonymized manner. Statistical analysis was performed using IBM SPSS Statistics program version 30.0.0.0 (172) [IBM Corporation, Armonk, NY, USA]. During the data analysis, we did not exclude patients whose data were missing.

Descriptive statistics were used to summarize the main sample characteristics. Categorical variables were described as counts (*n*) and proportions (%), while quantitative variables were summarized by measures of central tendency and dispersion, as means and standard deviations or medians and ranges, as appropriate. The Chi-square or Fisher’s exact tests, as appropriate, were used for comparison of proportions, while Student’s t-test or the one-way analysis of variance (ANOVA) or Kruskal–Wallis tests were used for comparison of means or medians, as adequate. All hypotheses tests were two-sided and a *p*-value <0.05 was considered statistically significant. Logistic regression was used to compute odds ratios (ORs) and their respective 95% confidence intervals (CIs) for the association between the level of surgical complexity and the occurrence of complications, considering all the variables associated with both. We conducted a sensitivity analysis, considering only clinically significant complications in relation to the complexity levels of the surgical techniques.

## Results

After undergoing breast-conserving surgery at our center, data from 304 female patients were analyzed consecutively. Only female patients were included, as data were collected consecutively and only this patient population underwent the procedures of interest. Figure 1 shows the distribution of patients across each surgical group, according to the novel classification system: levels 1, 2, 3, and 4 of complexity interventions were performed in 28 (9.2%), 121 (39.8%), 114 (37.5%), and 41 (13.5%) patients, respectively. The demographic and tumor characteristics are presented in Table 1. The mean age of patients was 59.8 years and the mean BMI was 27.3 kg/m^2^. All patients were Caucasians. Regarding the patients’ clinical history, 34 (11.2%) were smokers, 123 (40.5%) had hypertension, 46 (15.1%) had diabetes, 25 (8.2%) had undergone previous breast surgery, 6 (2.0%) had undergone previous chemotherapy and hormone therapy, and 8 (2.6%) had undergone previous chest wall radiation therapy. A total of 15 (4.9%) patients underwent primary chemotherapy.

**Table 1:** Patient and tumor characteristics [*n* = 304]

	Level of surgical complexity				
	1	2	3	4	*p*-Value
Age, years [mean (SD)]	66.9 (15.7)	61.2 (11.5)	57.8 (11.6)	60.0 (11.5)	**0.028**
*BMI, kg/m*^*2*^* [mean (SD)]*	26.7 (5.1)	26.9 (4.9)	28.5 (4.7)	25.8 (4.0)	**0.013**
<25	14 (13.0)	45 (41.7)	29 (26.9)	20 (18.5)	
≥25	14 (7.6)	72 (39.1)	79 (42.9)	19 (10.3)	0.058
Unavailable	0 (0.0)	4 (33.3)	6 (50.0)	2 (16.7)	
*Smoking*					0.089
No	25 (9.3)	112 (41.5)	101 (37.4)	32 (11.9)	
Yes	3 (8.8)	9 (26.5)	13 (38.2)	9 (26.5)	
*Diabetes mellitus*					0.09
No	21 (8.1)	98 (38.0)	103 (39.9)	36 (14.0)	
Yes	7 (15.2)	23 (50.0)	11 (23.9)	5 (10.9)	
*Hypertension*					0.855
No	16 (8.8)	69 (38.1)	71 (39.2)	25 (13.8)	
Yes	12 (9.8)	52 (42.3)	43 (35.0)	16 (13.0)	
*Previous breast surgery*					0.231
No	25 (9.0)	107 (38.4)	109 (39.1)	38 (13.6)	
Yes	3 (12.0)	14 (56.0)	5 (20.0)	3 (12.0)	
*Previous CT*					0.709
No	27 (9.1)	118 (39.6)	112 (37.6)	41(13.8)	
Yes	1 (16.7)	3 (50.0)	2 (33.3)	0 (0.0)	
*Previous chest wall RT*					0.456
No	27 (9.1)	116 (39.2)	112 (37.8)	41(13.9)	
Yes	1 (12.5)	5 (62.5)	2 (25.0)	0 (0.0)	
*Previous HT*					0.709
No	27 (9.1)	118 (39.6)	112 (37.6)	41(13.8)	
Yes	1 (16.7)	3 (50.0)	2 (33.3)	0 (0.0)	
*Breast cup size*^*a*^					**<0.001**
A, B	13 (11.6)	51 (45.5)	28 (25.0)	20 (17.9)	
>B	11 (7.4)	56 (37.8)	72 (48.6)	9 (6.1)	
Unavailable	4 (9.1)	14 (31.8)	14 (31.8)	12 (27.3)	
*Ptosis*^*a*^					**0.007**
0	1 (50.0)	0 (0.0)	1 (50.0)	0 (0.0)	
1	6 (7.9)	36 (47.4)	24 (31.6)	10 (13.2)	
2	11 (10.6)	42 (40.4)	43 (41.3)	8 (7.7)	
3	4 (8.0)	18 (36.0)	25 (50.0)	3 (6.0)	
Unavailable	6 (8.3)	25 (34.7)	21 (29.2)	20 (27.8)	
*Palpable mass*					**0.005**
No	9 (8.0)	59 (52.7)	35 (31.3)	9 (8.0)	
Yes	19 (10.0)	62 (32.6)	77 (40.5)	32 (16.8)	
Unavailable	0 (0.0)	0 (0.0)	2 (100.0)	0 (0.0)	
Size, mm [mean (SD)]	16.5 (7.8)	15.8 (8.5)	18.0 (12.0)	21.9 (12.4)	**0.048**
*BI-RADS*					**0.049**
2	1 (100.0)	0 (0.0)	0 (0.0)	0 (0.0)	
3	0 (0.0)	0 (0.0)	0 (0.0)	1 (100.0)	
4A	2 (15.4)	3 (23.1)	8 (61.5)	0 (0.0)	
4B	6 (11.8)	17 (33.3)	21 (41.2)	7 (13.7)	
4C	9 (7.6)	57 (47.9)	40 (33.6)	13 (10.9)	
5	10 (8.5)	43 (36.4)	45 (38.1)	20 (16.9)	
Unavailable	0 (0.0)	1 (100.0)	0 (0.0)	0 (0.0)	
*Location*					**0.002**
UOQ	11 (9.8)	44 (39.3)	43 (38.4)	14 (12.5)	
UQT	3 (9.7)	14 (45.2)	9 (29.0)	5 (16.1)	
UIQ	2 (4.3)	25 (53.2)	19 (40.4)	1 (2.1)	
IQT	2 (14.3)	8 (57.1)	3 (21.4)	1 (7.1)	
LIQ	1 (5.0)	8 (40.0)	7 (35.0)	4 (20.0)	
LQT	0 (0.0)	2 (20.0)	7 (70.0)	1 (10.0)	
LOQ	1 (4.3)	9 (39.1)	7 (30.4)	6 (26.1)	
OQT	2 (6.1)	7 (21.2)	15 (45.5)	9 (27.3)	
NAC	5 (38.5)	4 (30.8)	4 (30.8)	0(0.0)	
Unavailable	1 (100.0)	0 (0.0)	0 (0.0)	0(0.0)	
*Radiological appearance*					0.107
Nodule	24 (10.8)	91 (40.8)	83 (37.2)	25 (11.2)	
Microcalcifications	0 (0.0)	13 (44.8)	9 (31.0)	7 (24.1)	
Nodule and microcalcifications	3 (6.0)	16 (32.0)	22 (44.0)	9 (18.0)	
Unavailable	1 (50.0)	1 (50.0)	0 (0.0)	0 (0.0)	
*Focality*					0.164
Unifocal	27 (10.8)	100 (40.2)	90 (36.1)	32 (12.9)	
Multifocal	1 (1.8)	21 (38.2)	24 (43.6)	9 (16.4)	

Data are expressed as *n* (%) unless otherwise specified

*BMI* body mass index, *CT* chemotherapy, *RT* radiotherapy, *HT* hormone therapy, *BI-RADS* Breast Imaging Reporting and Data System, *IQT* inner quadrants transition, *LIQ* lower-inner quadrant, *LOQ* lower-outer quadrant, *LQT* lower quadrants transition, *OQT* outer quadrants transition, *UIQ* upper inner quadrant, *UOQ* upper outer quadrant, *UQT* upper quadrants transition, *NAC* nipple areola complex, *SD* standard deviation

^a^We considered Regnault’s classification for ptosis measurements, and the European Cup size classification for breast cup measurements

In 112/304 (36.8%) patients analyzed, breast cup size was either A or B, while 148 (48.7%) patients had a breast cup size larger than B. Data regarding breast cup size were unavailable for 44 (14.5%) patients. Ptosis grade was 0, 1, 2, or 3 in 2 (0.7%), 76 (25%), 104 (34.2%), and 50 (16.4%) cases, respectively; however, ptosis data from 72 (23.7%) patients were unavailable. A total of 190 (62.5 %) patients had a palpable mass. Older patients and those with a higher BMI preferably underwent less-complex techniques (*p* = 0.028 and *p* = 0.013, respectively).

Most patients with smaller cup sizes (A, B) underwent level 1 and 2 surgery techniques, while patients with bigger cup sizes (larger than B) preferably underwent level 3 techniques (*p* < 0.001). Patients with a higher ptosis grade (*p* = 0.007), a palpable mass (*p* = 0.005), larger tumor size (*p* = 0.048), and a higher Breast Imaging Reporting and Data System (BI-RADS) classification (*p* = 0.049) preferably underwent more complex procedures.

Among patients who had a tumor on the upper inner quadrant, 53.2% and 40.4% underwent level 2 and 3 procedures, respectively, with statistically significant differences in location of the tumor according to the complexity of the surgery. (*p* = 0.002).

The operative data and complications are presented in Table 2. The mean operative time, considering unilateral, bilateral, and symmetrization procedures, was 129.4 ± 55 min. More complex types of surgery led to an extended surgery duration, and this difference across surgery levels of complexity was statistically significant (*p* < 0.001).

**Table 2:** Operative data and complications [*n* = 304]

	Level of surgical complexity				
	1	2	3	4	*p*-Value
Operative time [mean (SD)]	90.0 (23.6)	95.33 (36.5)	156.5 (61.6)	150.6 (60.3)	**<0.001**
*Outpatient surgery*					0.058
No	19 (67.9)	64 (52.9)	53 (46.5)	15 (36.6)	
Yes	9 (32.1)	57 (47.1)	61 (53.5)	26 (63.4)	
No. of hospitalization days [median (range)]	2.0 (5.0)	1.0 (6.0)	1.0 (4.0)	1.0 (4.0)	0.637
Largest dimension, mm^b^	14.5 (20.0)	12.0 (39.0)	15.0 (125.0)	19.5 (71.0)	**<0.001**
[median (range)]					
Specimen weight, g^b^	28.5 (71.0)	38.0 (201.0)	68.0 (1013.0)	58.5 (155.0)	**<0.001**
[median (range)]					
*Complications*^a^					**<0.001**
No	25 (89.3)	93 (76.9)	69 (60.5)	22 (53.7)	
Yes	3 (10.7)	28 (23.1)	45 (39.5)	19 (46.3)	
*No. of complications*					**0.003**
None	25 (89.3)	93 (76.9)	69 (60.5)	22 (53.7)	
1	3 (10.7)	25 (20.7)	35 (30.7)	14 (34.1)	
≥2	0 (0.0)	3 (2.5)	10 (8.8)	5 (12.2)	
*Infection*					0.32
No	28 (100.0)	110 (90.9)	105 (92.1)	36 (87.8)	
Yes	0 (0.0)	11 (9.1)	9 (7.9)	5 (12.2)	
*Seroma*					**<0.001**
No	27 (96.4)	114 (94.2)	105 (92.1)	30 (73.2)	
Yes	1 (3.6)	7 (5.8)	9 (7.9)	11 (26.8)	
*Hematoma*					0.149
No	28 (100.0)	116 (95.9)	104 (91.2)	40 (97.6)	
Yes	0 (0.0)	5 (4.1)	10 (8.8)	1 (2.4)	
*Dehiscence*					**<0.001**
No	27 (96.4)	116 (95.9)	91 (79.8)	34 (82.9)	
Yes	1 (3.6)	5 (4.1)	23 (20.2)	7 (17.1)	
*Other complications*					0.994
No	27 (96.4)	116 (95.9)	109 (95.6)	39 (95.1)	
Yes	1 (3.6)	5 (4.1)	5 (4.4)	2 (4.9)	
*Clavien–Dindo*					**0.005**
0	25 (89.3)	93 (76.9)	71 (62.3)	23 (56.1)	
I	3 (10.7)	15 (12.4)	27 (23.7)	8 (19.5)	
II	0 (0.0)	10 (8.3)	11 (9.6)	3 (7.3)	
IIIA	0 (0.0)	3 (2.5)	3 (2.6)	5 (12.2)	
IIIB	0 (0.0)	0 (0.0)	2 (1.8)	2 (4.9)	
*Reoperation after a definitive diagnosis*					**0.021**
No	26 (92.9)	116 (95.9)	101 (88.6)	33 (80.5)	
Yes	2 (7.1)	5 (4.1)	13 (11.4)	8 (19.5)	
*Reoperation due to positive margins*					0.063
No	26 (92.9)	116 (95.9)	103 (90.4)	34 (82.9)	
Yes	2 (7.1)	5 (4.1)	11 (9.6)	7 (17.1)	
*Reoperation due to complications*					0.104
No	28 (100.0)	121 (100.0)	112 (98.2)	39 (95.1)	
Yes	0 (0.0)	0 (0.0)	2 (1.8)	2 (4.9)	
Duration of follow up, months [mean (SD)]	18.0 (7.3)	17.9 (6.0)	18.6 (5.6)	17.5 (6.6)	0.647

Data are expressed as *n* (%) unless otherwise specified

*SD* standard deviation

^a^Complications occurred within 30 days after surgery. ^b^The largest dimension and specimen weight were recorded, taking into consideration pathological results following analysis of the surgical specimen

A total of 95 (31.3%) patients had complications within 30 days after surgery: 25 (8.2%) had an infection, 28 (9.2%) had seroma, 16 (5.3%) had a hematoma, 36 (11.8%) had wound suture dehiscence, and 13 (4.3%) had other complications. A single complication was reported in 77 (25.3%) patients, while two or more complications were reported in 18 (5.9%) patients. An increasing proportion of patients with complications and more than one surgical complication was observed according to the increasing surgery complexity level (*p* < 0.001 and *p* = 0.003, respectively).

As the complexity of the surgical procedure increased, a higher incidence of seroma and dehiscence was observed (*p* < 0.001 and *p* < 0.001, respectively). Patient complications were classified according to the Clavien–Dindo classification: 53 (17.4%), 24 (7.9%), 11 (3.6%), and 4 (1.3%) were classified as level I, II, IIIA and IIIB, respectively. We concluded that more complex surgical procedures, classified as levels 3 and 4, resulted in higher Clavien–Dindo grades (*p* = 0.005).

A total of 28 (9.2%) patients had a reoperation, with an increasing trend across the surgery complexity levels (*p* = 0.021). In 25 cases (8.2%), patients required reoperation because of close or involved margins in final pathology, and in 4 cases (1.3%), reoperation was due to surgical complications; one patient required re-intervention due to both surgical complications and involved margins in the final pathology. Fifteen patients required surgical re-intervention due to complications, while a total of 4 (1.3%) patients required a reoperation under general anesthesia. The remaining complications, such as non-complicated infection, hematoma, and seroma, did not require any re-intervention. With a mean follow-up period of 18 months, 4 patients experienced locoregional recurrence of the disease. Patients with a higher BMI (>30; *p* = 0.042), larger breast cup size (*p* = 0.017), higher specimen weight (*p* < 0.001) and largest dimension (*p* = 0.040) experienced a higher frequency of complications (Table 3).

**Table 3:** Comparison of sociodemographic and clinical characteristics according to the occurrence of complications

	Complications		
	No [*n *= 209]	Yes [*n* = 95]	*p*-Value
Age, years [mean (SD)]	60.9 (12.5)	58.8 (10.9)	0.082
*BMI, kg/m*^*2*^* [mean (SD)]*	26.8 (4.7)	28.5 (4.9)	**0.004**
<25	82 (75.9)	26 (24.1)	**0.016**
≥25	116 (63.0)	68 (37.0)	
Unavailable	11 (91.7)	1 (8.3)	
*Smoking*			0.086
No	190 (70.4)	80 (29.6)	
Yes	19 (55.9)	15 (44.1)	
*Hypertension*			0.057
No	132 (72.9)	49 (27.1)	
Yes	77 (62.6)	46 (37.4)	
*Diabetes*			0.365
No	180 (69.8)	78 (30.2)	
Yes	29 (63.0)	17 (37.0)	
*Breast cup size*			**0.017**
A, B	88 (78.6)	24 (21.4)	
>B	92 (62.2)	56 (37.8)	
Unavailable	29 (65.9)	15 (34.1)	
Largest dimension, mm [median (range)]	14.0 (125)	16.0 (79)	**0.040**
Specimen weight, g [median (range)]	41.0 (1015.0)	65.0 (420.0)	**<0.001**

Data are expressed as *n* (%) unless otherwise specified

*BMI* body mass index, *SD* standard deviation

The odds of complications increased according to the level of surgical complexity, independently of both risk factors for complications and factors that influenced the surgery type selection. Considering that patients with seroma alone, classified as Clavien–Dindo 0 or I, were patients who did not have a clinically relevant complication, our results showed that the odds of developing a clinically important complication still increased according to the level of complexity. This effect was independent of other risk factors for complications and factors associated with selection of surgical technique (Table 4).

**Table 4:** Association between the level of surgical complexity and the occurrence of complications, as well as the occurrence of clinically relevant complications

	Complications				Relevant complications			
Level of surgical complexity	Crude OR (95% CI)	*p*-Value	Adjusted OR^a^ (95% CI)	*p*-Value	Crude OR (95% CI)	*p*-Value	Adjusted OR^a^ (95% CI)	*p*-Value
1	1 [ref]		1 [reference]		1 [reference]		1 [reference]	
2	2.51 (0.71–8.93)	0.156	8.71 (0.86–88.58)	0.068	3.22 (0.71–14.50)	0.128	5.40 (0.54–54.40)	0.153
3	5.43 (1.55–19.07)	**0.008**	13.42 (1.28–132.79)	**0.030**	7.87 (1.78–34.84)	**0.007**	11.51 (1.16–114.09)	**0.037**
4	7.20 (1.87–27.64)	**0.004**	34.55 (3.05–390.92)	**0.004**	6.04 (1.24–29.35)	**0.026**	15.53 (1.39–173.82)	**0.026**

*OR* odds ratio, *CI* confidence interval, *BMI* body mass index, *BI-RADS* breast imaging reporting and data system

^a^Adjusted for age, BMI, breast cup size and ptosis of the breast, palpable mass, specimen size, weight and location, and *BI-RADS* grade classification

## Discussion

This study was conducted due to the observed lack of standardized classification systems for BCOS correlated with morbidity. Thus, in accordance with international publications, there is an increasing interest in oncoplastic procedures and consensus for standardization is actively sought.^18,20,21^

Our novel classification system emerged from the need to standardize breast-conserving surgical procedures in our center and to group them in a way that allows for reproducible, periodic analysis of surgical morbidity. We found that current classification systems are often ambiguous in how they categorize specific procedures. Moreover, we believe it is more relevant to focus on the oncoplastic technique itself, rather than on the laterality (bilateral vs. unilateral), when assessing the independent influence of technique on morbidity. In this initial step, we deliberately chose to separate and analyze independent breast-conserving procedures from mastectomies and total breast reconstruction, as these represent fundamentally different groups in terms of patient selection and adjuvant treatment, particularly radiotherapy.

We compared our system with some of the most widely referenced existing classifications, including those proposed by Urban, Clough et al., Weber et al., and the ASBrS,^16–19^ noting that while these established systems offer valuable frameworks, they also present certain limitations when considered in light of the specific aims of our approach.

Urban proposed a three-tiered system encompassing low, moderate, and high complexity oncoplastic procedures.^16^ However, this classification conflates different surgical dimensions, such as laterality and reconstructive intent, without strict anatomical or technical definitions. It also includes mastectomy-based reconstructions alongside breast-conserving procedures, which limits its specificity and applicability when the focus is morbidity within conservative surgery.

Clough et al. introduced a binary system distinguishing between volume displacement and volume replacement techniques.^17^ While conceptually useful, this dichotomy does not capture the full gradient of technical complexity within each category and provides little guidance on how specific procedures should be stratified, resulting in variability in interpretation.

Weber et al. presented a descriptive framework that included a wide range of procedures, but did not propose a formal stratification by complexity.^18^ Although useful as a reference and decision-making guide, we believe that review lacks a reproducible structure for risk or morbidity stratification, which our system provides.

The ASBrS proposed a classification focused on standardizing terminology and procedural definitions.^19^ While comprehensive, this system does not stratify procedures by complexity or their potential morbidity.

In contrast, our proposed classification focused exclusively on breast-conserving oncoplastic procedures, enhancing both technical relevance and internal consistency. It establishes four distinct levels of complexity, defined not only by anatomical and technical criteria but also by the degree of tissue mobilization and parenchymal reshaping. It provides explicit assignment of common procedures to each level, thereby improving reproducibility and practical application. Furthermore, it is supported by a retrospective morbidity analysis demonstrating a significant association between classification level and complication rates.

Our retrospective cohort analyzed 304 patients consecutively operated at our breast center who underwent BCOS and whose personal, oncological, and surgical information was documented in clinical records. We aimed to validate a new classification system that allows to stratify different BCOS techniques, according to their level of complexity. We compared the characteristics of patients who underwent different surgical techniques, with increasing level of complexity, based on the predefined classification system (Fig. 1). The morbidity associated with each technique was assessed through postoperative complications, the Clavien–Dindo classification grade, and the need for re-intervention.

This study found that higher surgical complexity increases the odds of complications, independently of risk factors (BMI, breast cup size, specimen weight, tumor size) and surgery type selection. Both level 3 and 4 showed a statistically significant increase in the odds of having postsurgical complications. Furthermore, although there is a low rate of reoperation, it becomes progressively higher with the complexity level of the oncoplastic technique used. Therefore, we demonstrated that there is a statistically significant difference in morbidity due to surgery according to the different levels of complexity.

We conducted a sensitivity analysis, excluding from the complications group patients who had seroma as their sole complication. We observed that the association remained statistically significant, with an increased odds of developing complications persisting even when minor complications with limited impact on patient prognosis and follow-up were not considered. This analysis strengthens the study’s robustness as it demonstrates that the independent association remains valid with increasing surgical complexity, even when only considering complications with greater clinical impact.

We found that a patient’s age, BMI, breast characteristics, tumor size, presence of a palpable lump, tumor location, and BIRADS classification played a role in the choice of surgery performed. Our results show that more complex interventions are more time-consuming, reflecting the need for more resources and surgical skills. We can suppose that this can lead to more adverse effects associated with the intraoperative stress ambience that the patient experiences.^22^

We found that approximately 25% of patients experienced a single complication and approximately 6% had more than one complication. Regarding patient characteristics that are well-known factors that increase the risk of complications, we found a positive association between BMI and breast cup size, as well as the presence of complications.

Both the occurrence of complications and the number of complications were higher at increased levels of complexity. When it comes to specific complications, we observed that the occurrence of seroma and dehiscence was related to the complexity level of the technique. In level 3 and 4 procedures, a larger area is typically excised and a greater volume is displaced, resulting in increased unfilled space, which facilitates seroma formation. Additionally, these techniques subject the wound to greater tension, increasing the likelihood of dehiscence. However, no significant association was observed between the complexity level and the occurrence of infection, hematoma, and other complications alone.

Our analysis revealed that more complex oncoplastic techniques are associated with higher Clavien–Dindo grades (IIIA and IIIB), indicating an increased need for interventions under local anesthesia or even general anesthesia. These complications included infected fat necrosis or wound suture dehiscence, both observed more frequently in patients who had undergone techniques of higher complexity. These findings highlight how far complications can affect the normal postoperative course and their impact on patients’ quality of life, and how postsurgical surveillance should be tailored according to surgery type.

The implementation of this novel classification system aims to facilitate the scientific evaluation of oncoplastic procedures, therefore enabling surgeons to select a tailored procedure for each patient with more robust evidence, which allows for better planning and optimization of surgical techniques. Establishing reliable and well-defined comprehensive procedures is imperative to enhance aesthetic outcomes and patient quality of life, enabling objective comparisons and improvements in clinical practice.^23^

As this was a retrospective, single-center, observational study, there are some limitations that may have affected the results. The selection bias inherent in this type of study arises from factors associated with both patients and surgeons. For younger patients, surgeons tend to choose oncoplastic techniques to achieve better aesthetic outcomes. For older patients, surgeons tend to recommend non-oncoplastic techniques to mitigate the risk of adverse events associated with more extensive interventions.^15^ However, our results were not affected by selection bias based on patient age, as our system exclusively included conservative surgeries, encompassing both non-oncoplastic and oncoplastic techniques. Therefore, our findings remain independent of the surgeon’s selection prior to surgery.

The adjusted ORs reported in this study show wide confidence intervals, considering that level 1 complications occurred in only 10% of cases—the lower limit used for sample size calculation, which resulted in small subgroup sizes, decreased our sample power from 80% to approximately 60%. This could have been minimized with a larger sample size, yet it still adds robustness to the study.

Due to the study’s retrospective design, missing data could lead to an underestimation of associations and hinder the interpretation of analyses with missing data. There was limited information regarding breast cup size, breast ptosis, and BMI. Nevertheless, we did not exclude patients whose data were missing, in order to mitigate the bias associated with missing data.

The present study provides data from real-world patients, reflects the reality in a reference breast center in Portugal, and adds evidence suggesting that there are differences in the groups of surgeries analyzed regarding overall morbidity, which shows the relevance of this classification system. It aims to improve clinical practice, helping to individualize immediate postoperative care and surveillance and research on this matter, and ultimately better selecting patients to benefit from surgical outcomes and patient quality of life. Nevertheless, these findings must be interpreted with caution due to the limitations associated with observational data.

This study may serve as a potential starting point for future prospective studies, aimed at validating these findings. There is also room for further refinement of the classification system to enhance its applicability in clinical practice.

## Conclusions

This study demonstrates that the odds of having a postsurgical complication increase when the level of complexity of the BCOS increases, according to the novel classification system, highlighting the importance of adopting a classification system that clearly organizes techniques in a reproducible way to improve clinical practice.

## References

[1] Arnold M, Morgan E, Rumgay H, et al. Current and future burden of breast cancer: Global statistics for 2020 and 2040. *Breast*. 2022;66:15–23. 36084384 10.1016/j.breast.2022.08.010 PMC9465273

[2] Huang J, Chan PS, Lok V, et al. Global incidence and mortality of breast cancer: a trend analysis. *Aging (Albany NY)*. 2021;13(4):5748–803. 33592581 10.18632/aging.202502 PMC7950292

[3] Kashyap D, Pal D, Sharma R, et al. Global Increase in Breast Cancer Incidence: Risk Factors and Preventive Measures. *Biomed Res Int*. 2022;2022:9605439. 35480139 10.1155/2022/9605439 PMC9038417

[4] Keelan S, Flanagan M, Hill ADK. Evolving Trends in Surgical Management of Breast Cancer: An Analysis of 30 Years of Practice Changing Papers. *Front Oncol*. 2021;11:622621. 34422626 10.3389/fonc.2021.622621 PMC8371403

[5] Sakorafas GH. Breast cancer surgery–historical evolution, current status and future perspectives. *Acta Oncol*. 2001;40(1):5–18. 11321660 10.1080/028418601750070984

[6] Onitilo AA, Engel JM, Stankowski RV, et al. Survival Comparisons for Breast Conserving Surgery and Mastectomy Revisited: Community Experience and the Role of Radiation Therapy. *Clin Med Res*. 2015;13(2):65–73. 25487237 10.3121/cmr.2014.1245 PMC4504664

[7] Ng ET, Ang RZ, Tran BX, et al. Comparing Quality of Life in Breast Cancer Patients Who Underwent Mastectomy Versus Breast-Conserving Surgery: A Meta-Analysis. *Int J Environ Res Public Health*. 2019;16(24):4970. 31817811 10.3390/ijerph16244970 PMC6950729

[8] Thompson PW, Chatterjee A, Losken A. Standards in oncoplastic breast-conserving surgery. Annals of Breast Surgery 2021; 6.

[9] Kaufman CS. Increasing Role of Oncoplastic Surgery for Breast Cancer. *Curr Oncol Rep*. 2019;21(12):111. 31838584 10.1007/s11912-019-0860-9 PMC6911616

[10] Zucca-Matthes G, Manconi A, da Costa Viera RA, et al. The evolution of mastectomies in the oncoplastic breast surgery era. *Gland Surg*. 2013;2(2):102–6. 25083466 10.3978/j.issn.2227-684X.2013.05.03 PMC4115729

[11] Silverstein MJ, Mai T, Savalia N, et al. Oncoplastic breast conservation surgery: the new paradigm. *J Surg Oncol*. 2014;110(1):82–9. 24847860 10.1002/jso.23641

[12] Christiansen P, Carstensen SL, Ejlertsen B, et al. Breast conserving surgery versus mastectomy: overall and relative survival-a population based study by the Danish Breast Cancer Cooperative Group (DBCG). *Acta Oncol*. 2018;57(1):19–25. 29168674 10.1080/0284186X.2017.1403042

[13] Christiansen P, Mele M, Bodilsen A, et al. Breast-Conserving Surgery or Mastectomy?: Impact on Survival. *Ann Surg Open*. 2022;3(4):e205. 37600290 10.1097/AS9.0000000000000205 PMC10406082

[14] Duangkaew C, Somwangprasert A, Watcharachan K, et al. Comparison of Survival Outcomes of Breast-Conserving Surgery Plus Radiotherapy with Mastectomy in Early Breast Cancer Patients: Less Is More? *Cancers (Basel)*. 2025;17(4):591. 40002186 10.3390/cancers17040591 PMC11852543

[15] Oberhauser I, Zeindler J, Ritter M, et al. Impact of Oncoplastic Breast Surgery on Rate of Complications, Time to Adjuvant Treatment, and Risk of Recurrence. *Breast Care (Basel)*. 2021;16(5):452–60. 34720804 10.1159/000511728 PMC8543287

[16] de Andrade Urban C. New classification for oncoplastic procedures in surgical practice. *Breast*. 2008;17(4):321–2. 18485704 10.1016/j.breast.2007.11.032

[17] Clough KB, Kaufman GJ, Nos C, et al. Improving breast cancer surgery: a classification and quadrant per quadrant atlas for oncoplastic surgery. *Ann Surg Oncol*. 2010;17(5):1375–91. 20140531 10.1245/s10434-009-0792-y

[18] Weber WP, Soysal SD, Fulco I, et al. Standardization of oncoplastic breast conserving surgery. *Eur J Surg Oncol*. 2017;43(7):1236–43. 28214053 10.1016/j.ejso.2017.01.006

[19] Chatterjee A, Gass J, Patel K, et al. A Consensus Definition and Classification System of Oncoplastic Surgery Developed by the American Society of Breast Surgeons. *Ann Surg Oncol*. 2019;26(11):3436–44. 30977016 10.1245/s10434-019-07345-4

[20] Panhofer P, Ferenc V, Schutz M, et al. Standardization of morbidity assessment in breast cancer surgery using the Clavien Dindo Classification. *Int J Surg*. 2014;12(4):334–9. 24486930 10.1016/j.ijsu.2014.01.012

[21] Maliko N, Schok T, Bijker N, et al. Oncoplastic Breast Conserving Surgery: Is There a Need for Standardization? Results of a Nationwide Survey. *Breast Care (Basel)*. 2023;18(2):90–6. 37261127 10.1159/000528635 PMC10228254

[22] Cusack B, Buggy DJ. Anaesthesia, analgesia, and the surgical stress response. *BJA Educ*. 2020;20(9):321–8. 33456967 10.1016/j.bjae.2020.04.006 PMC7807970

[23] Weber WP, Soysal SD, El-Tamer M, et al. First international consensus conference on standardization of oncoplastic breast conserving surgery. *Breast Cancer Res Treat*. 2017;165(1):139–49. 28578506 10.1007/s10549-017-4314-5

